# *Bifidobacterium bifidum* Shows More Diversified Ways of Relieving Non-Alcoholic Fatty Liver Compared with *Bifidobacterium adolescentis*

**DOI:** 10.3390/biomedicines10010084

**Published:** 2021-12-31

**Authors:** Linlin Wang, Ting Jiao, Qiangqing Yu, Jialiang Wang, Luyao Wang, Gang Wang, Hao Zhang, Jianxin Zhao, Wei Chen

**Affiliations:** 1 State Key Laboratory of Dairy Biotechnology, Shanghai Engineering Research Center of Dairy Biotechnology, Dairy Research Institute, Bright Dairy & Food Co., Ltd., Shanghai 200436, China; wanglinlin@jiangnan.edu.cn; 2 State Key Laboratory of Food Science and Technology, School of Food Science and Technology, Jiangnan University, Wuxi 214122, China; janetine@163.com (T.J.); 6200113228@stu.jiangnan.edu.cn (Q.Y.); wjl120587@126.com (J.W.); ariluyao@foxmail.com (L.W.); wanggang@jiangnan.edu.cn (G.W.); zhang-hao61@jiangnan.edu.cn (H.Z.); chenwei66@jiangnan.edu.cn (W.C.); 3 School of Food Science and Technology, Jiangnan University, Wuxi 214122, China; 4 (Yangzhou) Institute of Food Biotechnology, Jiangnan University, Yangzhou 225004, China; 5 National Engineering Research Center for Functional Food, Jiangnan University, Wuxi 214122, China; 6 Wuxi Translational Medicine Research Center, Jiangsu Translational Medicine Research Institute, Wuxi Branch, Wuxi 214122, China

**Keywords:** non-alcoholic fatty liver disease, *Bifidobacterium adolescentis*, *Bifidobacterium bifidum*, gut microbiota, short chain fatty acids

## Abstract

The occurrence of non-alcoholic fatty liver disease (NAFLD) is closely related to intestinal microbiota disturbance, and probiotics has become a new strategy to assist in alleviating NAFLD. In order to investigate the effect of *Bifidobacterium* on NAFLD and the possible pathway, a NAFLD model was established by using a high-fat diet (HFD) for 18 weeks. Fourteen strains of *Bifidobacterium* were selected (seven *Bifidobacterium adolescentis* and seven *Bifidobacterium bifidum*) for intervention. The effects of different bifidobacteria on NAFLD were evaluated from liver cell injury, liver fat deposition, liver inflammatory state and liver histopathology, and were taken as entry points to explore the mitigation approaches of bifidobacteria through energy intake, lipid metabolism, glucose metabolism and intestinal permeability. The results showed that *Bifidobacterium* exerts species-specific effects on NAFLD. *B. bifidum* exerted these effects mainly through regulating the intestinal microbiota, increasing the relative abundance of *Faecalibaculum* and *Lactobacillus*, decreasing the relative abundance of *Tyzzerella*, *Escherichia-Shigella*, *Intestinimonas*, *Osillibacter* and *Ruminiclostridium*, and further increasing the contents of propionic acid and butyric acid, regulating lipid metabolism and intestinal permeability, and ultimately inhibiting liver inflammation and fat accumulation to alleviate NAFLD. *B. adolescentis* exerted its effects mainly through changing the intestinal microbiota, increasing the content of propionic acid, regulating lipid metabolism and ultimately inhibiting liver inflammation to alleviate NAFLD.

## 1. Introduction

Non-alcoholic fatty liver disease (NAFLD) is fatty degeneration caused by the excessive accumulation of triglyceride (TG) in the body’s liver cells. Its pathological feature is that the medium and large fat vacuoles in liver pathological sections are greater than 5%. With the adjustment of dietary structure, the age of patients with NAFLD is gradually expanded, and it occurs in all ages, including children. According to epidemiology statistics, NAFLD incidence increased year by year. As of 2016, the global incidence of NAFLD reached about 24% [1]. The incidence of NAFLD was about 29.81% in China in 2019 [2], and was equal to the incidence of metabolic diseases such as obesity and type II diabetes. Therefore, the cost of treating NAFLD-related diseases will also increase. It is estimated that the cost of NAFLD-related diseases in Europe alone may reach hundreds of billions of euros in 2016 [1].

In the human body, the liver plays the dual role of energy conversion station and detoxification device. Nutrients and exogenous compounds absorbed by the intestine enter the liver circulation along with the blood. The nutrients are metabolized by the liver to supply the energy needed by the whole body and exogenous compounds are usually detoxified and recirculated by the liver [3]. From the perspective of physiological function, the physiological state of the intestine determines the metabolic burden of the liver and the state of the liver also affects the operation of intestinal function. Numerous studies have shown that the occurrence of NAFLD is accompanied by the disturbance of intestinal microbiota [4,5,6]. Intestinal diseases (such as inflammatory bowel disease and Crohn’s disease) are closely related to the occurrence of liver diseases [7]. Letexier changed the microbiota by using prebiotics to reduce liver fat deposition [8]. These all indicate that intestinal microbiota may be an important vector for the formation of NAFLD. In recent years, domestic and foreign research shows that intestinal microbiota disorder mediated intestinal barrier damage and that metabolic disorder is the main cause of NAFLD. Vanessa transplanted feces from obese and leaner individuals into sterile mice, and mice that received feces from obese individuals also became obese, thus demonstrating the importance of intestinal microbiota in metabolism of the body [9]. In addition, Backhed took normal mice cecum contents daubed on the fur of germ-free mice; compared with the intervention mice, the other mice reduced their dietary intake but became fatter and developed energy metabolism disorder [10]. These not only confirmed the intestinal microbiota’s influence on the health of the host, but also made it clear that the gut bacteria can affect the host’s energy absorption and energy storage.

It is well known that probiotics are good regulators of intestinal microbiota, among which Bifidobacterium is one of the main sources of probiotics. *Bifidobacterium adolescentis* (*B. adolescentis*) and *Bifidobacterium bifidum* (*B. bifidum*) are the dominant bifidobacteria in the human intestinal microbiota, and are mainly colonized in the young. Former studies have found that *B. adolescentis* and *B. bifidum* play an important role in alleviating metabolic diseases [11,12]. At present, there are relatively few studies on the mitigation of NAFLD by probiotics, so the number of probiotics with the mitigation of NAFLD is also relatively small. Among the relevant research, we found that a large number of studies focused on single bacteria, which could not be compared between the same bacteria due to the differences in modeling methods, intervention time and measurement indicators. This would create obstacles to the analysis of rules. Based on the above difficulties, we selected seven strains of *B. adolescentis* and seven strains of *B. bifidum* to evaluate the effect of alleviating NAFLD, enrich existing resources and, finally, understand the mechanism through which bifidobacteria alleviates NAFLD.

## 2. Materials and Methods

### 2.1. Bifidobacteria Strains and Culture

Seven strains of *B. adolescentis* and seven strains of *B. bifidum* were obtained from the Food Biotechnology Centre of Jiangnan University. All the strains were cultured as mentioned above [11]. Before use, nine times volume of sterile distilled water was added to resuspend the strains, so the strain concentration was diluted to 1.0 × 10^9^ CFU/mL.

### 2.2. Animal Experiment Design

A total of 108 male C57BL/6J mice (4 weeks old) weighing 11–14 g were purchased from Shanghai Lingchang Biotechnology Co., Ltd. (Shanghai, China), and raised in an SPF-grade facility. After adaptive feeding for a week, the mice were randomly divided into 18 groups according to weight grading, with 6 mice in each group. The mice in the negative control group (NC) were fed a low-fat and low-sugar feed (TP23302, Trophic, Nantong, China), while the other mice were fed a 60% high-fat feed, which was optimized according to the Research Diets D12492 (TP23300, Trophic, Nantong, China). The group information was as follows: negative control group (NC) and model control group (HFD); metformin as a positive drug control group of hypoglycemic (MC); simvastatin as a positive drug control group of hypolipidemic (SC); and *B. adolescentis* group (Ba6, Ba7, Ba9, Ba11, Ba12, Ba13, Ba14) and *B. bifidum* group (Bb2, Bb2, Bb7, Bb8, Bb9, Bb10, Bb11) as different bifidobacteria treatment groups. The NC and HFD groups were administered 0.2 mL of 3% (*m*/*v*) sucrose solution every day, the MC group was administered 0.2 mL metformin (150 mg/kg·bw) every day, the SC group was administered 0.2 mL simvastatin (3 mg/kg·bw) every day, the intervention group was administered 0.2 mL bacteria suspension (5.0 × 10^8^ CFU/mL) every day, while the strains and drugs that were used were suspended in 3% (*m*/*v*) sucrose solution. The lavage and dietary interventions were sustained for 18 weeks. Mice in different groups were independently reared in IVC cages at 22 ± 2 °C and 55 ± 15% humidity. During the rearing process, light was strictly controlled day and night for 12 h, and irradiated feed and purified water were freely consumed. The sterilized clean cage boxes and bedding materials were replaced weekly and the health of the animals was observed daily. The ethical review number of the animal experiments is: JN. NO. 20190315C1280825 [12]. At 18 weeks, the feces of the mice were collected and stored in a refrigerator at −80 °C. After 12 h of fasting, the mice were weighed and injected with 1% pentobarbital sodium solution (50 mg/kg·bw) for anesthesia.

### 2.3. Determination of Biochemical Indexes in Serum

The serum levels of triglyceride (TG), total cholesterol (TC), high-density lipoprotein cholesterol (HDL-C), low-density lipoprotein cholesterol (LDL-C), alanine aminotransferase (ALT), aspartate aminotransferase (AST), cholinesterase (CHE), glucose (GLU) and free fatty acid (FFA) were determined using an automatic biochemical analyzer (Mindray, Shenzhen, China).

### 2.4. Quantification Cytokines in the Liver

The levels of interleukin-1β (IL-1β), tumor necrosis factor-α (TNF-α) and interleukin-6 (IL-6) in the liver were determined using an enzyme-linked immunosorbent assay (ELISA) kit (R&D Systems, Minneapolis, MN, USA).

### 2.5. Hepatic Histopathological Analysis

The preparation and observation of the liver sections were performed according to previous operations [11]; the liver scoring system was SAF, which was performed according to the method described by Bedossa [13].

### 2.6. Short-Chain Fatty Acids (SCFAs) Analysis in the Feces

The extraction and analysis of SCFAs in feces were described by Wang [14].

### 2.7. Intestinal Metagenome

DNA in feces was extracted with a MP Biomedicals DNA Rapid Extraction Kit and 16S rDNA sequencing and analysis were carried out according to the method described by Wang [15].

### 2.8. Statistics and Mapping

One-way ANOVA and correlation analyses were performed using SPSS (SPSS Inc., Chicago, IL, USA), GraphPad Prism6 and TBtools were used to create the graph. LEFSE analyses were completed online (http://huttenhower.sph.harvard.edu/galaxy/). All the experimental results were expressed as mean ± standard deviation (SD). Any two columns that do not contain the same lowercase letter (ABCDEF) in the same bar chart indicate statistical significance (*p* < 0.05).

## 3. Results

### 3.1. Bifidobacterium Exerts Species-Specific Effects on NAFLD

**Figure 1:**
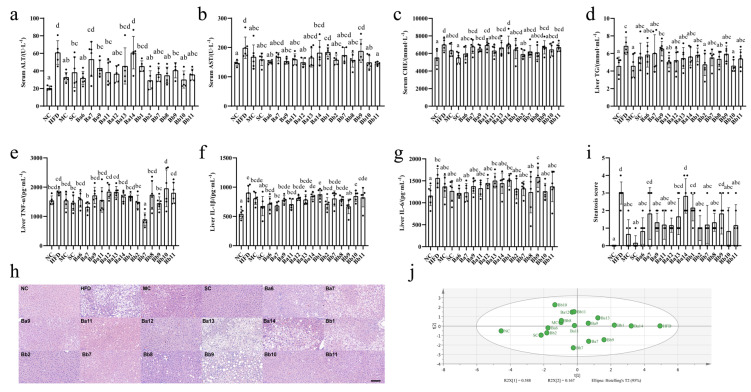
The effect of *Bifidobacterium* on liver injury in NAFLD mice. (**a**) ALT in serum; (**b**) AST in serum; (**c**) CHE in serum; (**d**) TG in liver; (**e**) TNF-α in liver; (**f**) IL-1β in liver; (**g**) IL-6 in liver; (**h**) Liver histopathology score, (**i**) Principal Component Analysis of each index of liver injury. (**j**) Principal component analysis of each index. Mean values with different letters (a–e) over the bars are significantly different (*p* < 0.05) according to Duncan’s multiple range test.

From the relevant detection indicators of NAFLD (liver cell injury, hepatic lipid accumulation, liver inflammation and liver pathology), we found that the mice in the HFD group displayed symptoms of NAFLD (Figure 1a–h).

On liver cell injury, the intervention of drugs and *Bifidobacterium* displayed different degrees of hindrance to the increase in ALT, AST and CHE. Among these, the serum ALT levels in the Ba7, Ba13 and Ba14 groups were not significantly different from the HFD group; in the other groups, they were significantly reduced (Figure 1a, *p* < 0.05). The serum AST levels in the Ba7, Ba13, Ba14, Bb1, Bb7 and Bb9 groups were not significantly different from the HFD group; in the other groups, they were significantly reduced (Figure 1b, *p* < 0.05). Simvastatin, SC, Ba6 and Bb2 intervention significantly decreased the concentration of serum CHE (Figure 1c, *p* < 0.05).

In terms of hepatic lipid accumulation, compared with HFD group, drug and strain intervention reduced the concentration of the liver TG to different extents, while in the metformin group, Ba11, Bb2, Bb8 and Bb10, the TG levels decreased significantly (Figure 1d, *p* < 0.05), to near the level of the NC group.

In terms of liver inflammation, compared with the HFD group, only the Ba7 and Bb7 groups significantly reduced their levels of TNF-α (Figure 1e, *p* < 0.05), while the Ba6, Ba7, Ba11, Bb2 and Bb9 groups significantly reduced their levels of IL-1β (Figure 1f, *p* < 0.05).

In terms of liver pathology, compared with the HFD group, the groups that received drug and strain intervention demonstrated some easing in their liver damage: intervention with four strains of *B. adolescentis* (Ba6, Ba9, Ba11 and Ba12) and five strains of *B. bifidum* (Bb2, Bb7, Bb8, Bb10 and Bb11) significantly reduced hepatic lipid droplets and the number of inflammatory cell infiltration lesions (Figure S1). The liver biopsy score showed that the HFD score was significantly higher than that of the NC group (Figure 1h, *p* < 0.05). Compared with the HFD, the scores for four strains of *B. adolescentis* (Ba6, Ba9, Ba11 and Ba12) and five strains of *B. bifidum* (Bb2, Bb7, Bb8, Bb10 and Bb11) decreased significantly (Figure 1h, *p* < 0.05). In the index of liver damage, according to the results of the PCA (Figure 1i), the first and second principal components can explain 75.5% of the indicator change; the NC and HFD groups were far apart on the first principal component, the drug and strain intervention groups were between the NC and HFD groups. Meanwhile, the intervention groups with three strains of *B. adolescentis* (Ba6, Ba11, Ba12) and five strains of *B. bifidum* (Bb2, Bb7, Bb8, Bb10 and Bb11) were closer to the NC group, This indicated that three strains of *B. adolescentis* (Ba6, Ba11, Ba12) and five strains of *B. bifidum* (Bb2, Bb7, Bb8, Bb10 and Bb11) exerted a significant alleviating effect on NAFLD.

### 3.2. Bifidobacterium Mainly Affected Fat Accumulation in NAFLD Mice

**Figure 2:**
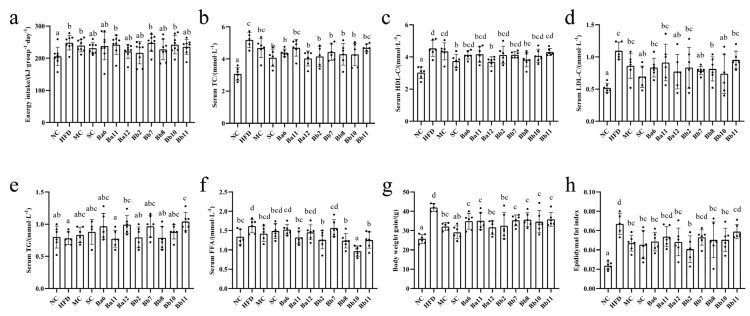
Effects of *Bifidobacterium* on energy intake and lipid metabolism in NAFLD mice. (**a**) energy intake; (**b**) TC in serum; (**c**) HDL-C in serum; (**d**) LDL-C in liver; (**e**) TG in serum; (**f**) FFA in serum; (**g**) body weight gain; (**h**) epididymal fat index. Mean values with different letters (a–d) over the bars are significantly different (*p* < 0.05) according to Duncan’s multiple range test.

The daily energy intake of the HFD group was 248.35 ± 22.73 kJ, which was significantly higher than that of the NC group (205.92 ± 28.37 kJ) (Figure 2a, *p* < 0.05). However, none of the intervention groups were significantly different from the HFD group (Figure 2a). These results indicated that the strain used in this study does not alleviate NAFLD by reducing energy intake.

**Figure 3:**
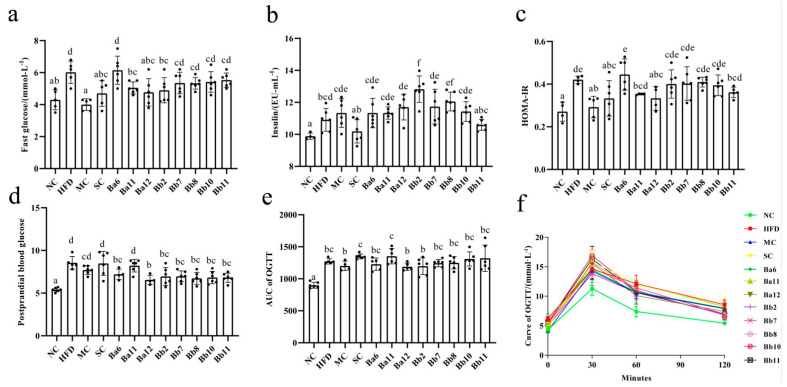
Effect of *Bifidobacterium* on blood glucose metabolism in NAFLD mice. (**a**) fasting glucose; (**b**) insulin; (**c**) HOMA-IR; (**d**) postprandial blood glucose; (**e**) AUC of OGTT; (**f**) curve of OGTT. Mean values with different letters (a–e) over the bars are significantly different (*p* < 0.05) according to Duncan’s multiple range test.

We measured the serum lipid indexes (serum TC, HDL-C, LDL-C, FFA and TG content) and fat accumulation index (weight gain and epididymal fat index) to clarify the effect of bifidobacteria on lipid metabolism disorders. As shown in Figure 3, the HFD group exhibited symptoms of lipid metabolism disorder (Figure 2b–h, *p* < 0.05). Compared with the HFD group, the levels of all the serum lipid indexes decreased significantly only in Bb8 group (Figure 2b–f, *p* < 0.05). Compared with the HFD group, the body weight gain was significantly lower in the drugs and bifidobacteria intervention groups (*p* < 0.05), while the epididymal fat index decreased significantly in the Ba6, Ba12, Bb2, Bb8 and Bb10 groups (*p* < 0.05). These results illustrated that bifidobacteria relieved NAFLD mainly focused on fat accumulation.

### 3.3. In the Relief of NAFLD, B. adolescentis Mainly Focused on Regulating Fasting Blood Glucose, While B. bifidum Focused on Postprandial Blood Glucose

Compared with the NC group, the fasting blood glucose, insulin, HOMA-IR and AUC of OGTT levels in the HFD group were significantly increased (Figure 3a–c, *p* < 0.05), indicating that NAFLD mice featured the characteristics of glucose metabolism disorder. Compared with HFD, the fasting blood glucose decreased significantly in the MC, SC, Ba11 and Ba12 groups (Figure 3a, *p* < 0.05) and significantly increased in the Bb2 and Bb8 groups (Figure 3a, *p* < 0.05). HOMA-IR reduced significantly in the MC, SC and Ba12 groups (Figure 3c, *p* < 0.05). The postprandial blood glucose concentration in the Ba6, Ba12, Bb2, Bb7, Bb8, Bb10 and Bb11 intervention groups were significantly decreased (Figure 3d, *p* < 0.05). The OGTT results showed that glucose concentration was at a normal level at 0 min of glucose loading, all the mice reached the maximum value at 30 min of glucose loading and the glucose level gradually decreased at 60 min and 120 min. At the 120th min, the glucose concentration in the HFD group was higher than that in the NC group (Figure 3f). There were no significant differences between the bifidobacterial intervention groups and the HFD group in the level of AUC of OGTT (Figure 3e). These results showed that in the relief of NAFLD, *B. adolescentis* mainly focused on regulating fasting blood glucose, while *B. bifidum* focused on postprandial blood glucose.

### 3.4. Bifidobacterium Affects Intestinal Permeability in NAFLD Mice

**Figure 4:**
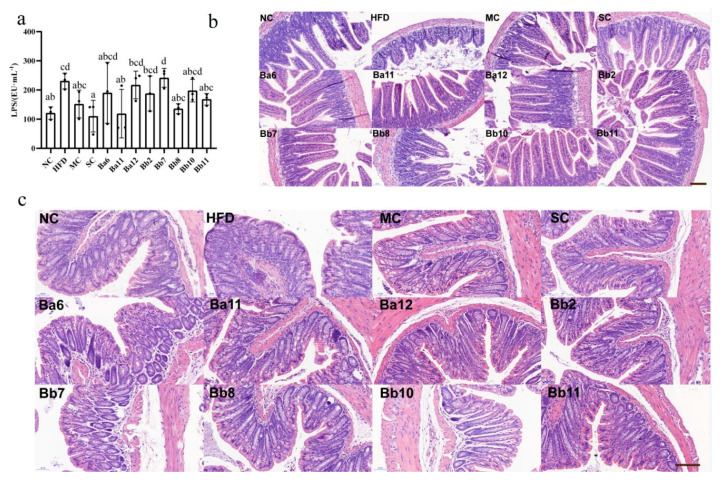
Effect of *Bifidobacterium* on intestinal permeability of NAFLD mice. (**a**) the concentration of serum LPS; (**b**) the ileal histopathology in NAFLD mice treated with different bifidobacteria; (**c**) the colon histopathology in NAFLD mice treated with different bifidobacterial. Scale bar = 100 μm. Mean values with different letters (a–d) over the bars are significantly different (*p* < 0.05) according to Duncan’s multiple range test.

The concentration of LPS in the HFD group was 208.19 ± 36.74 Eu/mL, which was significantly higher than that in the NC group (120.87 ± 21.27 Eu/mL), indicating that the long-term continuous intake of HFD promoted the increase of intestinal permeability (Figure 4a, *p* < 0.05). Compared with the HFD group, the serum LPS concentration in MC, SC, Ba11, Bb8 and Bb11 groups was significantly decreased (Figure 4a, *p* < 0.05), indicating that intervention with Ba11, Bb8 and Bb11 could improve intestinal permeability.

In order to clarify the improvement of intestinal permeability by effective *Bifidobacterium* further, pathological observations were performed on the ileum (Figure 4b) and colon (Figure 4c) sections of the mice. In the NC group, ileum tissue structure was complete, mucosal thickness was normal, and the villi in the small intestine were smooth and evenly distributed. There was a large number of goblet cells in the small intestine, which ensured the absorption and immune function of the ileum. Compared with NC group, the mucous membrane layer of the HFD group was thinner, the cells of the submucosa were distributed more sparsely, the intestinal villus density was reduced, the structure of the intestinal villus was significantly shorter and bulking deformation occurred, a few hairs broke and the surface was damaged, and the number of goblet cells decreased. In addition, part of the lamina propria formed inflammatory infiltrates and gathered a large number of inflammatory cells, showing that long-term, continued HFD promotes structural damage to the ileum mucosa. Compared with the HFD group, drug and *Bifidobacterium* interventions showed different status in different aspects. In the MC group, the length and density of the villi in the small intestine were increased and the number of goblet cells was increased. Inflammatory infiltration was present but improved, while the length of the villi in the SC group was improved, but the density of the villi decreased and the deformation of the villi was not significantly improved. In all the bifidobacteria intervention groups, the length and density of the villi in the small intestine were restored, the number of goblet cells was significantly increased and the number of inflammatory infiltrating lesions was decreased, indicating that the intervention of the strain exerted a repairing effect on the ileum mucosal barrier.

The pathological results of the colon (Figure 4c) showed that the mucosal surface of the NC group was smooth, without damage, while the number of crypts in the mucosal layer was large and the structure was normal; the crypts contained a large number of goblet cells, which ensured the secretion of mucus (Figure 4c). Compared with the NC group, the mucosal surface of the HFD group was slightly damaged, local inflammatory infiltration occurred in the mucosal layer, crypt depth was shortened, part of crypt structure was destroyed and the number of goblet cells decreased, indicating that long-term continuous intake of HFD promoted damage to the colon mucosa. Compared with the HFD group, the intervention group showed a repairing effect on the colonic mucosa in different aspects. Different interventions can reduce damage to the mucosal surface and the inflammatory infiltration of the mucosal layer. The crypt length and the number of cup cells in the MC, SC, Ba11, Bb8 and Bb11 groups increased, indicating that intervention with drugs and *Bifidobacterium* can repair the intestinal mucosal damage caused by the long-term intake of HFD.

### 3.5. The Effect of B. bifidum on SCFAs Was Greater than That of B. adolescentis

**Figure 5:**
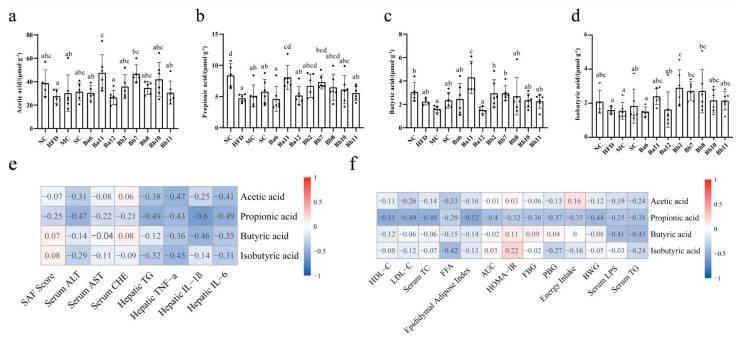
Effects of *Bifidobacterium* on SCFAs in NAFLD mice. (**a**) acetic acid; (**b**) propionic acid; (**c**) isobutyric acid; (**d**) butyric acid; (**e**) the correlation analysis of SCFAs and hepatic damage; (**f**) the correlation analysis of SCFAs and alleviation pathways. Mean values with different letters (a–d) over the bars are significantly different (*p* < 0.05) according to Duncan’s multiple range test. Cell injury indexes: serum ALT, serum AST and serum CHE; hepatic lipid accumulation index: hepatic TG. Liver inflammation indexes: hepatic TNF-α, IL-1β and IL-6. Liver lipid metabolism indexes: HDL-C, LDL-C, serum TC, serum TG, FFA. Epididymal fat index and body weight gain. Sugar metabolism indexes: AUC, HOMA-IR, FBG, PB.; Intestinal permeability index: serum LPS.

Compared with the NC group, the concentrations of acetic acid, propionic acid, isobutyric acid and butyric acid in the HFD group were decreased (Figure 5a–d), while the concentration of propionic acid was significantly decreased (Figure 5b, *p* < 0.05), indicating that the long-term continuous intake of HFD can reduce the production of intestinal SCFAs. Compared with HFD group, the *B. adolescentis* intervention group increased the concentration of propionic acid, while *B. bifidum* mainly increased the levels of propionic- and butyric acid (Figure 5).

### 3.6. B. adolescentis and B. bifidum Had Different Ways to Relieve NAFLD

Figure 5e,f shows the correlation analysis results of the SCFA levels, NAFLD evaluation indicators and mitigation pathway indicators. The results showed that the content of propionic acid was negatively correlated with liver lipid accumulation (hepatic TG) and liver inflammation indexes (TNF-α, IL-1β and IL-6), while the content of butyric acid was negatively correlated with liver inflammation (Figure 5e). On the mitigation pathway, indexes related to energy intake, lipid metabolism, glucose metabolism, and intestinal permeability were more correlated with propionic acid than other SCFAs and propionic acid was strong negatively correlated with lipid metabolism, while butyric acid was strong negatively correlated with liver intestinal permeability and liver lipid accumulation (r < −0.4), suggesting that propionic acid might mitigate NAFLD mainly by regulating lipid metabolism, while butyric acid relieved NAFLD mainly by regulating intestinal permeability.

In conclusion, *B. bifidum* mainly reduced liver lipid metabolism and intestinal permeability by increasing the content of propionic acid and butyric acid in feces and ultimately inhibited liver inflammation and fat accumulation to alleviate NAFLD. *B. adolescentis* mainly reduced liver lipid metabolism by increasing the content of propionic acid in feces and ultimately inhibited liver inflammation to alleviate NAFLD.

### 3.7. B. bifidum Exerted a Significant Impact on the Composition of the Intestinal Microbiota of NAFLD Mice

In order to observe the changes to the intestinal microbiota, six phyla were obtained after sequence analysis and annotation of all the samples: Firmicutes, Bacteroidetes, Tenericutes, Verrucomicrobia, Actinobacteria and Proteobacteria. Compared with the NC group, the Firmicutes and Actinobacteria of the HFD group were decreased, while the Tenericutes, Verrucomicrobia and Proteobacteria were increased (data not shown).

**Figure 6:**
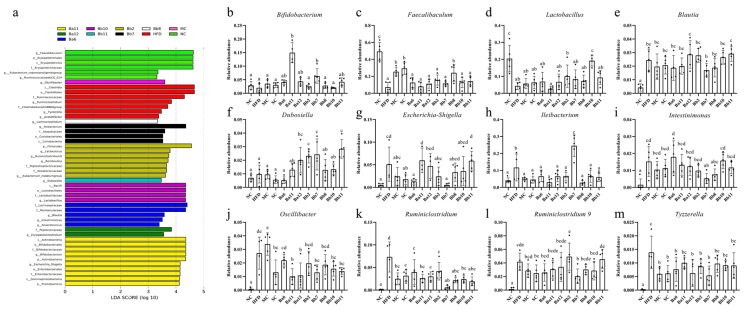
Effect of *Bifidobacterium* on intestinal microbiota of NAFLD mice. (**a**) the LEfSe analysis of gut microbiota after intervention with bifidobacteria in NAFLD mice; (**b**) *Bifidobacterium*; (**c**) *Faecalibaculum*; (**d**) *Lactobacillus*; (**e**) *Blautia*; (**f**) *Dubosiella*; (**g**) *Escherichia-Shigella*; (**h**) *Ileibacterium*; (**i**) *Intestinimonas*; (**j**) *Oscillibacter*; (**k**) *Ruminiclostridium*; (**l**) *Ruminiclostridium 9*; (**m**) *Tyzzerella.* Mean values with different letters (a–e) over the bars are significantly different (*p* < 0.05) according to Duncan’s multiple range test.

In order to clarify the changes to the intestinal microbiota caused by different interventions further, an LEfSe analysis was conducted for different classifications of intestinal microbiota (Figure 6a). Except for the SC group, all the groups featured their own differential genera; any genus that was more than 1% in the differential genera was classified and analyzed separately. The abundance of *Bifidobacterium*, *Lactobacillus* and *Faecalibaculum* in the HFD group was lower than in the NC group (Figure 6b–d), and the abundance of *Lactobacillus* and *Faecalibaculum* in the HFD group was significantly decreased (Figure 6c,d, *p* < 0.05). The intervention with metformin, simvastatin and eight strains of *Bifidobacterium* could increase the content of *Bifidobacterium*; the levels in the Ba11 and Bb7 groups were significantly higher than in the other intervention groups (Figure 6b, *p* < 0.05). The intervention with metformin, simvastatin, Ba6, Ba12, Bb2, Bb7, Bb8, Bb10 and Bb11 could increase the abundance of *Faecalibaculum* and *Lactobacillus* (Figure 6c,d).

The abundances of *Blautia*, *Escherichia-Shigella*, *Ileibacterium*, *Insominimonas*, *Oscillibacter*, *Ruminiclostridium*, *Ruminiclostridium 9* and *Tyzzerella* in the HFD group were significantly higher than in the NC group (Figure 6e–m, *p* < 0.05). The relative abundances of *Ruminiclostridium*, *Tyzzerella*, *Oscillibacter* and *Ileibacterium* decreased in almost all the *Bifidobacterium* intervention groups (Figure 6h,j,k,m), while the abundances of *Escherichia-Shigella* and *Intestinimonas* decreased mainly in the *B. bifidum* intervention groups (Figure 6g,i). The metformin and simvastatin intervention groups decreased their abundances of *Blautia*, *Escherichia-Shigella*, *Ileibacterium*, *Insominimonas*, *Oscillibacter, Ruminiclostridium*, *Ruminiclostridium 9* and *Tyzzerella* (Figure 6e–m). In conclusion, the effect of *B. bifidum* on the structure of intestinal microbiota of NAFLD mice was greater than that of the *B. adolescentis* intervention group.

### 3.8. The Formation of NAFLD Was Related to Intestinal Microbiota

**Figure 7:**
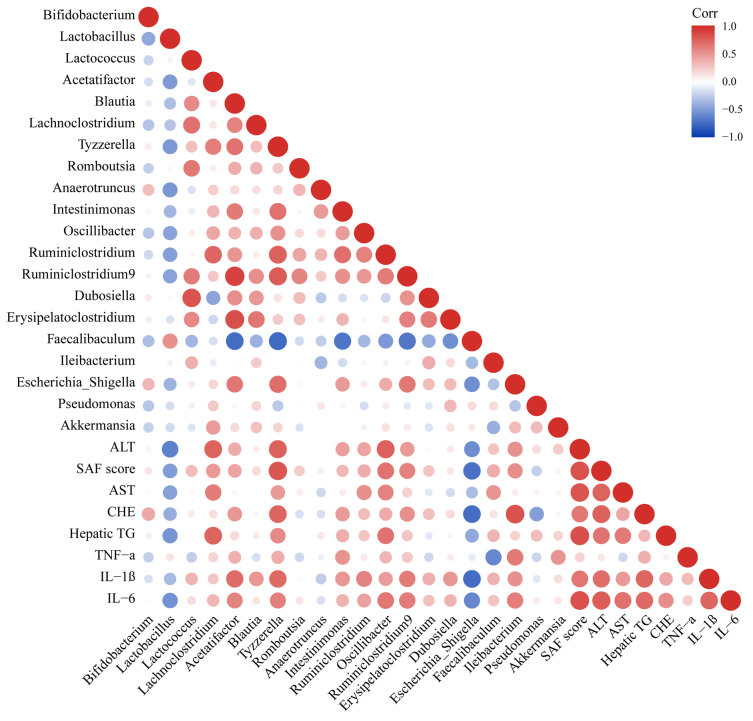
Correlation analysis between intestinal microbiota and liver injury.

In order to explore the role of differential genera in the formation of NAFLD, a correlation analysis was conducted between the relevant indicators of NAFLD (liver cell injury, hepatic lipid accumulation, liver inflammation and liver pathology) and the intestinal microbiota (Figure 7). According to the results, the liver cell injury indices the liver pathology score displayed a strong positive correlation with *Acetatifactor*, *Escherichia_Shigella*, *Intestinimonas*, *Oscillibacter*, *Ruminiclostridium*, *Ruminiclostridium 9* and *Tyzzerella* and negatively correlated with *Faecalibaculum* and *Lactobacillus* (|r| > 0.4). Hepatic lipid accumulation presented a strong positive correlation with the abundance of *Ruminiclostridium* and *Tyzzerella* (r > 0.4), and a strong negative correlation with the abundance of *Faecalibaculum* and *Lactobacillus* (r < −0.4). There was a strong positive correlation between the indexes of liver inflammation and the abundances of *Blautia*, *Erysipelatoclostridium*, *Escherichia-Shigella*, *Intestinimonas*, *Oscillibacter*, *Ruminiclostridium*, *Ruminiclostridium 9* and *Tyzzerella* (r > 0.4) and a strong negative correlation with *Faecalibaculum* and *Lactobacillus* (r < −0.4). These results indicated that the formation of NAFLD was related to intestinal microbiota.

### 3.9. Specific Intestinal Microbiota Was Related to Lipid Metabolism or Intestinal Permeability

**Figure 8:**
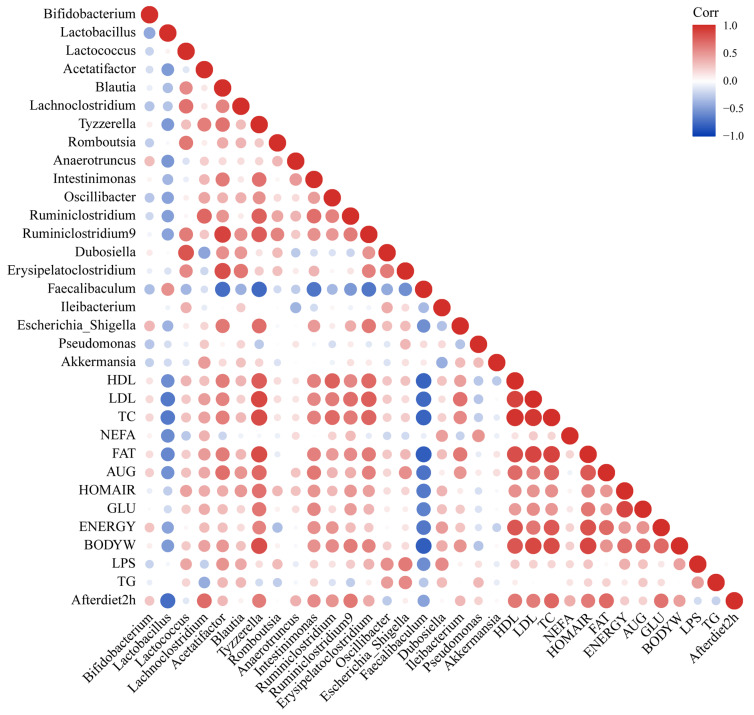
Correlation analysis of intestinal microbiota and other influencing factors.

In order to explore the role of intestinal microbiota in the related pathways (energy intake, lipid metabolism, sugar metabolism and intestinal permeability) for alleviating NAFLD, we established a study on the correlation between intestinal microbiota and mitigation pathways (Figure 8). The correlation showed that the relative abundances of *Lactobacillus* and *Faecalibaculum* were strongly negatively correlated with liver lipid metabolism indexes (HDL-C, LDL-C, TC, FFA, epididymis fat index and body weight gain) (r < −0.6), while the relative abundances of *Blautia*, *Intestinimonas*, *Oscillibacter*, *Ruminiclostridium*, *Ruminiclostridium 9* and *Tyzzerella* were strongly positive correlation with lipid metabolism (r > 0.4) (Figure 8). The relative abundances of *Blautia*, *Ileibacterium*, *Erysipelatoclostridium* and *Dubosiella* were strongly positively correlated with intestinal permeability. These results indicated that changing the abundance of different bacteria (such as *Lactobacillus* and *Faecalibaculum*) could regulate lipid metabolism or intestinal permeability in mice and ultimately alleviate NAFLD.

### 3.10. Changing the Abundance of Intestinal Microbiota That Featured Different Correlations with Propionic and Butyric Acid Could Alleviate NAFLD

**Figure 9:**
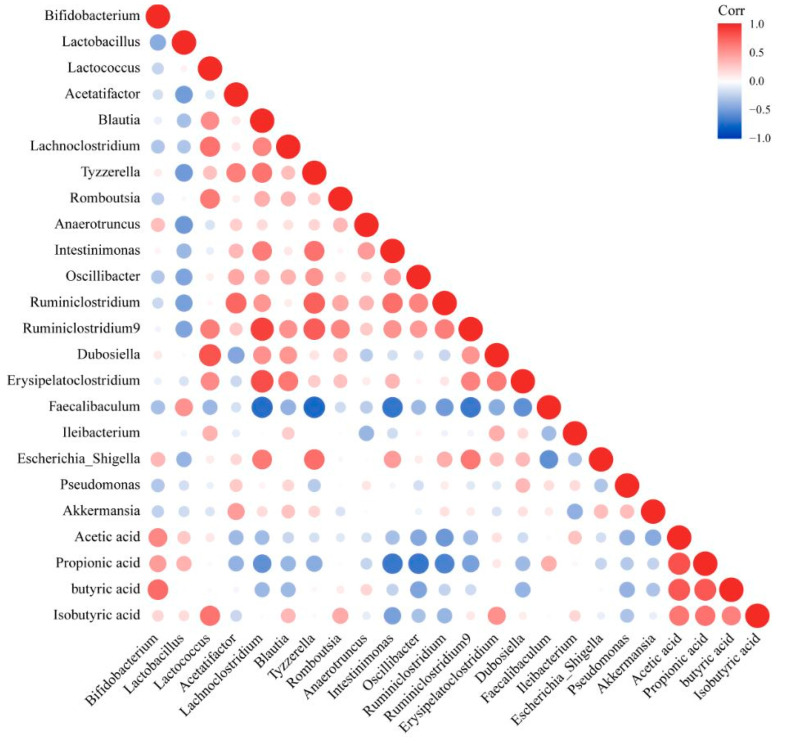
Correlation analysis between intestinal microbiota and SCFAs.

In order to explore the influence of the intestinal microbiota on its metabolites (SCFAs), we established a correlation between the intestinal microbiota and SCFAs (Figure 9). The content of acetic acid showed a moderately positive correlation with the abundances of *Bifidobacterium* (0.4 < r < 0.6) and a moderately negative correlation with the abundances of *Oscillibacter*, *Ruminiclostridium* and *Akkermansia* (−0.6 < r < −0.4) (Figure 9). There was a positive correlation between propionic acid and *Bifidobacterium*, *Lactobacillus* and *Faecalibaculum* and a negative correlation with *Blautia*, *Tyzzerella*, *Intestinimonas*, *Osillibacter*, *Ruminiclostridium 9* and *Ruminiclostridium* (Figure 9). There was a strong positive correlation between isobutyric acid and *Lactococcus*, a medium positive correlation with *Romboutsia* and *Dubosiella* and a medium negative correlation with *Intestinimonas* (Figure 9). There was a strong positive correlation between butyric acid and *Bifidobacterium* and a negative correlation with *Blautia*, *Oscillibacter and Erysipelatoclostridium* (Figure 9).

The combination of the results of the correlation between the intestinal microbiota and NAFLD detection indicators, NAFLD mitigation pathways and SCFAs levels in feces showed that an increase in the relative abundance of *Lactobacillus* and *Faecalibaculum*, which were positively correlated with propionic acid and a decrease in the relative abundance of *Blautia*, *Erysipelatoclostridium*, *Intestinimonas*, *Oscillibacter*, *Ruminiclostridium*, *Ruminiclostridium 9* and *Tyzzerella*, which were negatively correlated with propionic and butyric acid, could alleviate NAFLD.

In conclusion, the combination of all the above results indicated that *B. bifidum* mainly acted through regulating the intestinal microbiota, increasing the relative abundance of *Faecalibaculum* and *Lactobacillus*, decreasing the relative abundance of *Tyzzerella*, *Escherichia-Shigella*, *Intestinimonas*, *Osillibacter* and *Ruminiclostridium* and further increasing the contents of propionic acid and butyric acid, regulating lipid metabolism and intestinal permeability and ultimately inhibiting liver inflammation and fat accumulation to alleviate NAFLD. *B. adolescentis* mainly acted through changing the intestinal microbiota, increasing the content of propionic acid, regulating lipid metabolism and ultimately inhibiting liver inflammation to alleviate NAFLD.

## 4. Discussion

Intestinal microbiota disturbance is an important factor affecting the formation of NAFLD, so regulating intestinal microbiota to change intestinal metabolites became a new intervention strategy for the treatment of NAFLD [16]. In recent years, many reports have shown that *Bifidobacterium* exerts a good alleviating effect on NAFLD. The intervention with *B. Bifidum* BGN4 significantly alleviated the liver tissue damage induced by high-fat and high-sugar diets [17]. *B. Bifidum* BGN4 significantly improved the excessive liver fat deposition induced by persistent HFD [18] and *B. Bifidum* ATCC SD6576 reduced liver damage in NAFLD patients significantly [19]. *B. adolescentis* 15,705 alleviated the liver injury induced by western diets by reducing NF-kB pathway activation and lipid peroxidation [20]. Similarly, the present study showed that three *B. adolescentis* (Ba6, Ba11 and Ba12) and five *B. bifidum* strains (Bb2, Bb8, Bb7, Bb10 and Bb11) exerted a significant mitigation effect on persistent liver injury induced by HFD.

This paper evaluated the effects of *Bifidobacterium* on energy intake, fat metabolism, glucose metabolism, intestinal permeability, changes in intestinal microbiota and changes in microbial metabolites in alleviating NAFLD. It is well known that high energy intake is a key factor affecting the formation of NAFLD in mice [21]. Multiple studies have shown that probiotics can reduce the energy intake of the host in a variety of ways. Khalili proved that they could not only reduce energy intake significantly but also improve insulin resistance through the intragastric administration of lactic acid bacteria in insulin-resistant mice [22]. Cristina observed that *Bifidobacterium* and *Lactobacillus* can reduce energy intake by affecting GHSR-related signals and thereby reduce weight gain [23]. Multiple studies have shown that probiotics intake can affect energy intake by regulating the expression of appetite related signaling factors (ghrelin receptor, mTOR, and casein, etc.) in the body. However, unfortunately, the bifidobacteria in this paper failed to block the formation of NAFLD through this pathway.

The regulation of metabolic disorders is another important way in which probiotics alleviate NAFLD. In the regulation of lipid metabolism disorder, Khan found that plant seeds with lipid-lowering effects were effective at relieving liver damage caused by hyperlipidemia [24]. Jia also suggested that polysaccharides, which exert significant effects on reducing hematic fat, can effectively improve liver damage caused by continued HFD intake [25]. Furthermore, our results also showed that bifidobacteria, which can alleviate liver injury, could reduce the level of lipid accumulation in the serum and/or the fat accumulation in the liver. Ota proved that insulin resistance preceded the formation of NAFLD and alleviated the symptoms of NAFLD by improving insulin resistance significantly [26]. Rosenzweig also showed that improved glucose tolerance was associated with hepatic steatosis and that it exerted an inhibitory effect on hepatic steatosis by improving glucose tolerance [27]. Similarly, Mihailović showed that probiotics could regulate glucose metabolism disorders and positive effects were achieved in the intervention of liver injury caused by insulin resistance with probiotics [28]. Our results also showed that most of the bifidobacteria with the ability to alleviate liver injury offer certain advantages in regulating glucose metabolism disorders.

Liver injury is caused by metabolic disorders and intestinal microbiota disorders. The intervention of probiotics in metabolic diseases mainly regulates the metabolism of the body and repairs the intestinal barrier by regulating the intestinal microbiota. The intestinal barrier, which includes the intestinal microbial barrier, intestinal chemical barrier and intestinal physical barrier, which cooperate with each other to achieve protection, is the protective cover through which the body prevents the entry of exogenous pathogenic factors. Intestinal microbiota disturbance is the expression of intestinal microbial barrier disruption. High-fat diets promote the disturbance of the intestinal microbiota and induce the decreased secretion of antimicrobial peptide (α-defensiin, RegIII protein), leading to the decreased antimicrobial activity of the intestinal mucosa [29], which in turn leads to the excessive growth of some bacteria, causes further damage to the intestinal physical barrier and accelerates the formation of NAFLD [30,31]. Intestinal microbiota dysregulation induces the increased activity of myosin light chain kinase in the intestine, which reduces the expression of tight junction protein and leads to damage to the intestinal physical barrier [32]. Moreover, an imbalance in the intestinal microbiota leads to increased endogenous alcohol production, which destroys the physical barrier of the intestinal tract and increases intestinal permeability further [33], so that endotoxin and alcohol in the intestinal tract can directly act on the liver to cause damage [34]. Our results showed that intervention with bifidobacteria could change the intestinal microbiota of NAFLD mice, mainly manifested as a decrease in the abundance of bacteria genera with a strong positive correlation with liver injury and an increase in the abundance of bacteria genera with a strong negative correlation with liver injury. In terms of intestinal permeability, we observed that some strains significantly reduced the content of LPS in serum, indicating that intervention with *Bifidobacterium* reduced the production of LPS or repaired the intestinal barrier, reduced the entry of LPS into the blood and further reduced inflammatory reaction. The results of the correlation between the intestinal microbiota and LPS indicated that intestinal microbiota was closely related to the content of LPS, and regulating intestinal microbiota effectively reduced the content of LPS in serum. Therefore, for LPS-mediated diseases, regulating intestinal microbiota through probiotics was a good choice.

SCFAs are one of the main metabolites of intestinal microbiota and exert a regulatory effect on host metabolism. At present, there is no accurate positioning of the role of SCFAs in NAFLD, and most studies had shown that SCFAs could improve NAFLD. SCFAs activated GPCR in the intestine to increase the expression of fat oxidation-related genes (HSL, CPT1 and CPT2) and thereby reduced fat accumulation [35,36]. Acetic acid could activate GPR41/43 and MAPKs signals, propionic acid could affect the liver fat synthesis in order to reduce the accumulation of fat in the liver [37] and butyric acid could activate receptor γfactor-1 by improving the peroxidase in skeletal muscle and brown fat, thereby improving mitochondrial function to affect glucolipid metabolism in body [38]. The results of this paper showed that the content of propionic acid was negatively correlated with liver lipid accumulation (Hepatic TG) and liver inflammation indexes (TNF-α, IL-1β and IL-6), while the content of butyric acid was negatively correlated with liver inflammation. In the mitigation pathway, indexes related to energy intake, lipid metabolism, glucose metabolism, and intestinal permeability were more correlated with propionic acid than other SCFAs, while propionic acid was strongly negatively correlated with lipid metabolism and butyric acid was strong negatively correlated with liver intestinal permeability and liver lipid accumulation. This suggested that propionic acid might mitigate NAFLD, mainly by regulating lipid metabolism, while butyric acid relieved NAFLD mainly by regulating intestinal permeability.

## 5. Conclusions

This article evaluated the effects of different bifidobacteria on NAFLD from liver cell injury, liver fat deposition, liver inflammation and liver histopathology. These effects were taken as entry points through which to explore the mitigation approaches of bifidobacteria through energy intake, lipid metabolism, glucose metabolism, intestinal permeability, intestinal microbiota and its metabolites. The results showed that three strains of *B. adolescentis* and five strains of *B. bifidum* exert significant mitigative effects on NAFLD caused by high-fat diets. *B. bifidum* exerted its effects mainly through regulating the intestinal microbiota, increasing the relative abundance of *Faecalibaculum* and *Lactobacillus*, decreasing the relative abundance of *Tyzzerella*, *Escherichia-Shigella*, *Intestinimonas*, *Osillibacter* and *Ruminiclostridium* and further increasing the contents of propionic acid and butyric acid, regulating lipid metabolism and intestinal permeability and ultimately inhibiting liver inflammation and fat accumulation to alleviate NAFLD. *B. adolescentis* exerted its effects mainly through changing the intestinal microbiota, increasing the content of propionic acid, regulating lipid metabolism and ultimately inhibiting liver inflammation to alleviate NAFLD.

## Data Availability

Data are available from the authors upon reasonable request.

## Supplementary Materials

The following are available online at www.mdpi.com/article/10.3390/biomedicines10010084/s1. Figure S1: Pathological section of liver.
