# Supplementary figures and images for: Bifidobacterium bifidum Shows More Diversified Ways of Relieving Non-Alcoholic Fatty Liver Compared with Bifidobacterium adolescentis
Source: Biomedicines. 2021 Dec 31;10(1):84. doi: 10.3390/biomedicines10010084 (PMC8772902; doi:10.3390/biomedicines10010084)

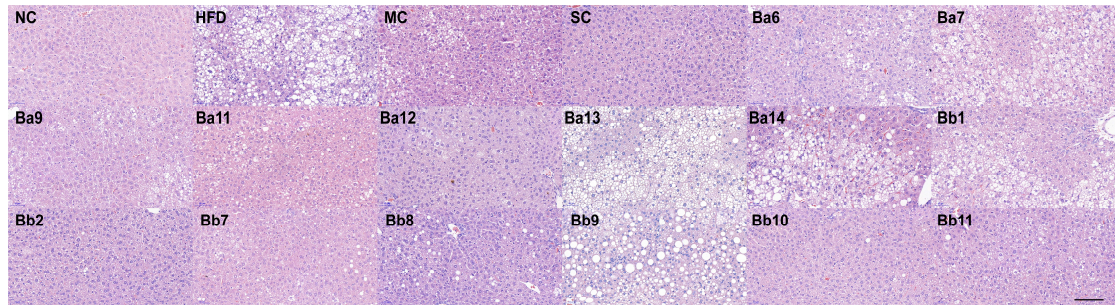

Figure S1. Pathological section of liver  
Scale bar = 100 $\mu$ m.

Supplement: Supplementary file 1 [file biomedicines-10-00084-s001.zip › biomedicines-1482045-supplementary.pdf]
